# 92180 Prevalence of Clostridioides difficile strains found in Texas soil

**DOI:** 10.1017/cts.2021.466

**Published:** 2021-03-30

**Authors:** Amina R. Zeidan, Kelsey Strey, Michelle N. Vargas, Kelly R. Reveles

**Affiliations:** University of Texas at Austin College of Pharmacy, UT Health San Antonio

## Abstract

ABSTRACT IMPACT: This work investigates C. difficile strains in soil as a potential exposure for gut colonization and community-acquired infection of C. difficile. OBJECTIVES/GOALS: Identifying environmental sources of C. difficile can inform how non-hospital reservoirs can potentially contribute to C. difficile exposure and subsequent gastrointestinal colonization. The objective of the study was to identify C. difficile and toxin genes across various soil sources. METHODS/STUDY POPULATION: This was a cross-sectional study utilizing soil samples obtained throughout Texas, USA. All samples were collected between August and November of 2019 and 2020. Samples were taken from human and animal high contact areas, such as recreational parks. Samples were stored at -80oC until processing. DNA extractions were performed using the DNeasy Powersoil Pro Kit (Qiagen) per manufacturer’s instructions. Real-time PCR was also performed on extracted DNA using the Microbial DNA qPCR Multi-Assay Kit for Clostridium difficile Pathogenicity (Qiagen) for the identification of C. difficile, toxin A (TcdA), and toxin B (TcdB) genes. RESULTS/ANTICIPATED RESULTS: A total of 137 soil samples including dry dirt, sand, and wet soil near water sources were collected and processed for the presence of C. difficile. These included samples from parks and trails (42.3%), water sources (36.5%), and other public spaces (21.2%). C. difficile was identified in 59 (43.1%) soil samples: 6 (4.4%) with Toxin A and 2 (1.5%) with toxin B production. C. difficile was most prevalent among samples taken from parks and trails (50.0%), followed by other public spaces (48.3%), and water sources (32.0%). The median (IQR) Cq value for the C. difficile gene was 39.24 (33.45-40.47) among samples that tested positive. DISCUSSION/SIGNIFICANCE OF FINDINGS: We identified a high prevalence of Clostridioides difficile in soil samples, though toxin gene detection prevalence was low. Future studies will analyze other sources, including water and varying surface samples to obtain a comprehensive view of C. difficile in the environment.

